# Adduction laryngeal dystonia: proposal and evaluation of nasofibroscopy

**DOI:** 10.1016/S1808-8694(15)30987-3

**Published:** 2015-10-19

**Authors:** Noemi Grigoletto De Biase, Paula Lorenzon, Mariana Dantas Aumond Lebl, Marina Padovani, Ingrid Gielow, Glaucya Madazio, Miriam Moraes

**Affiliations:** a PhD. Associate Professor – Speech and Hearing School - Pontifícia Universidade Católica de São Paulo. MD, collaborator at the Department of Otorhinolaryngology/Head and Neck Surgery – Voice and Larynx Lab – Federal University of São Paulo – Paulista School of Medicine; b MD. Otorhinolaryngologist and postgraduate student (MS) of Otorhinolaryngology – Federal University of São Paulo – Paulista School of Medicine (UNIFESP-EPM); c MD. MS in Otorhinolaryngology– Federal University of São Paulo – Paulista School of Medicine (UNIFESP-EPM); d Speech and Hearing Therapist. MS in Human Communications Sciences – Paulista School of Medicine (UNIFESP-EPM); e Speech and Hearing Therapist. PhD in Human Communications Sciences – Paulista School of Medicine (UNIFESP-EPM). Head of the Rehabilitation Department – Head and Neck Surgery/Voice Lab - UNIFESP-EPM; f Speech and Hearing Therapist. MS in Communications Sciences – Paulista School of Medicine (UNIFESP-EPM). PhD Student in Human Communications Sciences (UNIFESP-EPM); g Speech and Hearing Therapist, Expert in Voice – CEV (Voice Study Center). Collaborator at the Voice and Larynx Lab. Federal University of São Paulo – Paulista School of Medicine. Department of Otorhinolaryngology – Voice and Larynx Lab

**Keywords:** neurological disease, dystonia, glottal incompetence, vocal fold

## Abstract

Dystonias are organic central motor processing disorders characterized by involuntary muscular contractions or incontrollable spasms induced by task-specific movements. Adduction laryngeal dystonias present with important speech impairments, with inappropriate spasms and abrupt voice breaks. The diagnosis is based on clinical features, evaluation by a speech therapist and transnasal fiber optic laryngoscopy.

**Aim:**

Our objective is to propose and evaluate a task-oriented transnasal fiber optic laryngoscopy protocol, which shows the spasms, and propose maneuvers that reduce or make them disappear, in order to facilitate the diagnosis.

**Methods:**

transversal study. Analysis of the transnasal fiber optic laryngoscopy records of 15 patients with adductor laryngeal dystonia using the proposed protocol.

**Results:**

most of the speech and non-vocal tasks allowed us to identify the spasms and reduce or make them disappear. We propose the exclusion of two of the maneuvers that don't bring new data to the evaluation.

**Conclusion:**

the protocol was useful for the evaluation of the patients, showing changes in muscle behavior in the structure under investigation.

## INTRODUCTION

Dystonias are central motor processing organic disorders characterized by involuntary muscle contractions or uncontrollable spasms during an activity^1^, ^2^. These abnormal movements may be sustained for a variable amount of time, from one second to minutes and may occur in any part of the body; they are clearly not psychogenic in origin; however, they can get worse with fatigue, stress and emotions^3^. Dystonias have an unknown cause, although most authors think that the base ganglions are involved^4^. The classification of dystonias is usually related to the muscle group involved, being focal when it affects a specific muscle group, and generalized when it affects a great number of muscle groups. Between both types we find the segmentary type, which affects muscle groups which are close to each other in proximity. In children, the symptoms are of focal onset, followed by its spread to other body parts, while in adults, the symptoms usually remain focal and frequent in the head and neck^1^ - more rare focal forms involve the larynx intrinsic muscles. As far as the latter is concerned, the following types are described: adduction, abduction and respiratory^5^, ^6^, ^7^. The respiratory is the less frequent one and also the more concerning one, since it causes respiratory restriction of varied degrees, without dysphonia during speech^3^, ^8^. Abduction larynx focal dystonia is not very common and spasms occurs during speech formation on the posterior crycoarytenoid muscles. With this there is air escaping during sound production, translated by intermittent blowing voice in chained speech, more rare forms involve the larynx intrinsic muscles.

Focal adduction dystonia is more frequent. In this type there is a strong contraction of adducting muscles during speech formation, in other words, hyper adduction is innadequate^3^. In this type, voice is tense/strangled, with frequent sound breaks and clear vocal strain. The involvement varies and may seriously impair communication. Usually, there is little alteration in laughter, singing, whisper and falsetto^6^. The breaks occur when the vocal cords coming together is so intense that they do not allow air to pass through. The strongest contraction occurs when the vocal folds are adducted, that is during sound production, and they are more evident in words that start by a vowel. It may be followed by constant larynx tremor alone or involving pharyngeal muscles, or from the so called dystonic tremor seen only during speech production^4^, ^5^. Since there is no specific examination, diagnosis is based on clinical signs: hearing-perceptive voice assessment and laryngoscopy, specially through flexible optical fiber nasofibroscope^3^, ^10^. Adduction larynx dystonia must be specially differentiated from the skeleton-muscles tension syndromes and some cases of psychogenic dysphonia which bear resemblance in chained speech. Voice acoustic analysis objectively translates audible voice patterns and allows for the identification of voice tension and breaks. Electromyography may be useful in diagnostic confirmation, and the most frequently found signs are a sudden and periodic increase in electrophysiological potentials of the thyroarythenoid muscle and extension on pre and post-phonatory electrical activity^11^. Exam by nasofibrolaryngoscopy, more physiologically compatible than the telescopic exam, allows for the execution and evaluation of tasks that show clearly the clinical characteristics of adduction laryngeal dystonia.

Thus, our goal is to propose and assess a protocol of nasofibroscopy that uses tasks that show spasms and tasks that reduce them or make them disappear, in order to facilitate analysis and diagnosis.

## MATERIALS AND METHODS

A protocol of nasofibroscopy exam for the assessment of the palate, pharynx and larynx (Attachment 1), performed in 15 patients with adduction laryngeal dystonia, with diagnosis by audible-perceptive analysis, anterior nasofibrolaryngoscopy, electromyography and improvement after the injection of Botulin toxin in the vocal fold. Nasal-laryngeal fibroscopy was carried out after the patients signed an informed consent, and a Machida ENT-30 PIII device was used; the exam was recorded on a videocassette tape for later analysis which was carried out by three otorhinolaryngologists. The study was carried out in the Larynx and Voice outpatient ward of the Federal University of São Paulo - Paulista School of Medicine. The nasal-laryngeal fibroscopy exam was carried out at least six months after the injection of the Botulin Toxin and after the patient and the authors have observed a return of the tense/strangled characteristic in the patient's voice quality through audible-perceptive analysis. The nasofibroscope was introduced through either the right or the left nostril, and its tip placed close to the choana, in order to assess the palate. The analysis counted on more or less low tone emissions, with and without glottal adduction participation and during rest and swallowing. We also requested the patients to produce phrases. Afterwards, the device was placed on the rhinopharynx in order to observe the movement of the pharynx during rest and during the utterance of low and high sounds. Most of the assessment was carried out with the tip close to the larynx, in such a position that allowed the glottis to be seen. The patients were asked to utter low and high sounds, sound production with and without glottal adduction, phrases with a predominance of sonorous sounds, whispered voice, high intensity sound production and unusual tasks, and also, other non-phonatory sounds such as inhaling sound production, whistling and sniffing (Attachment 1). The exams were recorded for later analysis under normal and slow speeds, by three otorhinolaryngologists with experience in laryngology and dystonias, with agreement among the observers.

## RESULTS AND COMMENTS

In assessing the palate, all the patients presented complete closure of the pharyngeal sphincter. The assessment during phrases uttering was made difficult because of the spasm and did not add data in relation to the other tasks. Most of the patients did not show palate tremor during rest, only two of them did. The uttering of vowels “é”, “i” and “u” allows us to see differences in spasm intensity according to pitch variations. The uttering of sounds “s” and “z” allowed the assessment of a reduction or even disappearance of spasms during blunt uttering in adduction dystonias.

As to the pharynx, no patient had rest spasms and during the production of vowel “é”, in six patients we observed contractions of the pharynx walls.

Evaluation during inhaling did not show epiglottis or larynx movements, and such investigation will be useful in cases of respiratory dystonia. Laryngeal tremor during rest was seen in one case.

In assessing the larynx we noticed spasms during the “é” vowel sound in the habitual tone of voice, with disappearance or reduction with high glissing for most of the patients studied. Only three patients did not show any change, although in three other patients there was no change, three other could not gliss. We observed that our patients can not always do the ascending or the descending glissing sound. The increase in fundamental frequency with the utterance of the hyperacute “i” was possible with all of them, and only in two of them we did not see spasms reduction or disappearance. Thus, there was a difference in relation to the presence or reduction of spasms in all the patients during hyperacute sound production, except in two, and such task is useful due to its easy performance and spasms modification. The highest sounds, specially in falsetto, are uttered with a slight separation of the vocal folds, and this reduces spasm stimulus.

The utterance of phrases with a predominance of sonorous sounds followed by the phrase with blunt sounds allowed us to see a reduction of spasms in the blunt utterances. The comparison shows the difference, not always seen in chained speech. During whispering, the spasm disappeared in six patients, reduced in two, and the others were not able to produce the whisper and only repeated the phrase at a lower intensity, keeping the spasms. During whispering, since the vocal folds do not touch each other, we were able to see a spasm reduction or even disappearance. The utterance of the word “Gol” at a high intensity did not contribute to the assessment because it allows for “pitch” and intensity variations in a very short word. We could notice spasms in 7 patients, just like it happened with the utterance of the “é”, reduction in 3 patients and disappearance in the others, in whom we noticed high utterances.

In the tasks - inhaling phonation, whistling and sniffing - no patients had spasms. Such movements are not the ones commonly used for phonation and, therefore, should not trigger spasms, which depend on habitual phonation. It is likely that the brain circuit used for these tasks be different from the one used for phonation and be intact.

Thus, the protocol proved to be useful in the assessment of patients with laryngeal dystonia, bearing tasks that allow us to show the presence of spasms and tasks that show its reduction or even disappearance. Some tasks do not prove useful, and we propose their exclusion, thus making the assessment faster and more objective - they are: velopharyngeal closure investigation, including the use of phrases, during swallowing and the utterance of the word “Gol”.

## CONCLUSION

The protocol was useful in the assessment of patients, showing a change in the behavior of the muscle groups studied according to the tasks performed.

Attachment 1 Universidade Federal de São Paulo/UNIFESP

Department of Otorhinolaryngology

Laryngology and Voice

Joint Scale for Laryngeal Dystonia Assessment

Nasofibroscopy Assessment Protocol

Name:

Date:

General impression:

Functional assessment of the soft palate

**Table:** 

Tasks	Comments
Rest	

Swallowing	

É- I -U	

SSS-ZZZ	

Papai pediu pipoca para Pedro	

Mamãe comeu mamão	

Um homem e uma mulher viram um anjo voando	

O sapo saltou o sapato

Assessment of non-phonatory tasks, speech and voice

**Table:** 

Tasks	Comments
rest	

Long “eh”	

“eh” ascending gliss	

“ih” Hyperacute	

Inhaling phonation	

Snif-snif	

whistle	

whisper “Shh! The baby is asleep”	

Shout “Gol!”	

Repeat: “Um homem e uma mulher viram um anjo voando.”	

Repeat: “O sapo saltou o sapato”

Adapted from the Joint Scale of Spasmodic Dysphonia Assessment (USDRS)

Stewart CF et al. Adductor spasmodic dysphonia: standard evaluation of symptoms and severity. J Voice 1997;11(1):95-103.
